# Effect of *Acacia mearnsii* forage or tannin extract on rumen dry matter and crude protein degradation

**DOI:** 10.1111/jpn.14033

**Published:** 2024-08-09

**Authors:** Lindokuhle C. Mhlongo, Piers Kenyon, Ignatius V. Nsahlai

**Affiliations:** ^1^ Department of Animal Science University of the Free State Bloemfontein South Africa; ^2^ Department of Animal and Poultry Science University of KwaZulu‐Natal Scottsville Pietermaritzburg South Africa; ^3^ Ntlangwini Makhoba Farming (Pty) Ltd, Makhoba Land Swartberg South Africa

**Keywords:** condensed tannins, in sacco, non‐conventional feeds

## Abstract

This study investigated rumen degradation kinetics of dry matter (DM) and crude protein (CP) in compound feed with different tannin extract inclusions and *Acacia mearnsi* forage (AMF) relative to dairy feeds (perennial ryegrass+white clover mixture pasture, maize silage, lucerne hay and *Themeda triandra* hay). The compound feed had 0.75%, 1.5% and 3% tannins extract inclusions while the control was a commercial compound feed. Triplicates of each feed per incubation period were incubated in two fistulated Jersey cows for 0, 6, 24, 48, 72, 96 and 120 h, resulting in six replicates per feed. Tannin extract inclusions in compound feed only affected (*p* < 0.05) the (*a*) fraction, degradation rate and potential degradability (PD) for DM degradation, and affected (*p* < 0.05) the (*a*) and (*b*) fractions, as well as PD for CP degradation. The (*a*) fraction and degradation rate for DM degradation changed linearly (*p* < 0.05). The (*a*) fraction, PD and effective degradability (ED) of DM degradation changed quadratically (*p* < 0.05). Except for the degradation rate, the feed type affected (*p* < 0.05) the degradation parameters in both DM and CP degradations. For DM and CP degradations, (*a*) fraction was similarly the least in *Themeda triandra* hay and AMF but similarly the highest in maize silage, perennial ryegrass+white clover mixture pasture and lucerne hay. The (*b*) fraction was the least in AMF for both DM and CP degradations but the highest for pasture's DM degradation and similarly the highest in maize silage, lucerne hay and *T. triandra* hay for CP degradation. The PD was the least in AMF for both DM and CP degradations and similarly the highest in pasture DM but similarly the highest in perennial ryegrass+white clover mixture pasture, maize silage and lucerne hay for CP degradation. Furthermore, the ED was the lowest in AMF and the highest for perrenial ryegrass + white clover mixture pasture for DM degradation and same trend was observed for CP degradation whereby perrenial ryegrass + white clover mixture pasture, maize silage and lucerne had the highest ED. Digestible undegraded protein was the highest in AMF and similarly the least in dairy feeds. Tannin source inclusion in ruminant diets should be moderate to prevent rumen DM or CP degradation limitation.

## 1 INTRODUCTION

Dairy production requires the availability of quality feeds throughout the year to maintain high milk production. Dry seasons are associated with limited feed quality. For this reason, potentially cheap feeds are needed as supplements for dairy cows during dry periods. Unconventional feeds rich in crude proteins have the potential of being dairy protein supplements for their high protein content even during dry seasons (Basha et al., 2015).

The nutrient degradability in the rumen affects the productivity of dairy cows (Putri et al., 2021) as it determines animal performance through feed intake (Riasi et al., 2008). Tannins are common in unconventional feeds and decrease nutrient intake by reducing nutrient digestibility at certain inclusions in diets (Riasi et al., 2008). Therefore, unconventional feeds need to be studied for their rumen degradability to avoid deleterious inclusions of unconventional feeds on ruminant performance (Kamalak et al., 2005).

Tannins complex with proteins in the rumen and the resulting complex reverses post‐rumen, which promotes milk production (Riasi et al., 2008). However, at deleterious tanniferous feed inclusions, the tannin complex may not reverse in the small intestines when the tannin inclusions exceed 5% dietary inclusions, which decreases milk performance due to decreased protein digestibility (Dentinho et al., 2019). Furthermore, tannins inhibit fibre digestion by binding fibre, which reduces nutrient intake and digestibility. Therefore, studying the effect of different tannin inclusions and tanniferous feeds compared to conventional dairy diets on the rumen degradation characteristics of proteins can provide insight on how tannins can be added to dairy feeds without depressing nutrient intake and digestibility.

*Acacia mearnsii* forage (AMF) commonly known as Black wattle is a possible alternative feed supplement or tannin extract source to enhance animal performance (Lamba et al., 2014). The tannin extract inclusion of this feed in dairy feeds has conflicting results on feed intake, CH_4_ emissions and milk production (Alves et al., 2017). The inconsistent results of tannin extract or tanniferous feeds on animal performance could be attributed to inadequate understanding of the effect of tannins on the rumen degradation kinetics (Perna Junior et al., 2022; Tseu et al., 2020). Dietary tannin level has been reported to influence rumen degradation kinetics (Neumann et al., 2020). The tannin effect on degradation kinetics changes depending on the tannin source (Basha et al., 2014; Melaku et al., 2010).

Therefore, investigating AMF as an individual feed in relation to conventional dairy feeds or AMF tannin extract inclusions improves the understanding of their tannin effect on ruminal degradation kinetics. The objective of this study was to investigate the dry matter (DM) and crude protein (CP) rumen degradation kinetics of different tannin extract inclusions in compound feed and AMF relative to dairy feeds (maize silage, lucerne, *T. triandra* hay and perennial ryegrass+white clover mixture pasture). It was hypothesized that rumen degradation kinetics would be decreased with increased tannin extract inclusions in compound feed and decreased in AMF relative to dairy feeds.

## 2 MATERIALS AND METHODS

### 2.1 Treatments and data collection

The feeds used in the study were collected from the Springfontein dairy farm, Kokstad, South Africa (30°15′52.0″S, 29°10′15.0″E S and 1450 m altitude). Regular feeds of the Springfontein dairy farm including maize silage, lucerne hay, *T. triandra* hay and perennial ryegrass+white clover mixture pasture were assessed for their rumen degradability in comparison to that of AMF (leaves and twigs) as a nonconventional feed (Table 1). Additionally, compound feed of different tannin extract inclusion was assessed for their rumen degradability kinetics in comparison to the regular commercial compound feed (15% CP from AFGRI, South Africa) fed to Springfontein dairy farm cows as a control (Table 2). In this study, commercial compound feed served as control compound feed (0% tannin extract) due to their wide acceptance in the dairy industry and to being fed daily to dairy cows of the Springfontein dairy farm from which feeds of this study were sourced. The control compound feed lacked ingredient information, and the experimental compound feed consisted of custom compound feed containing (0.75%, 1.5% or 3%, as fed basis) AMF tannin extract processed at MIMOSA extract company (NTE House, Redlands Estate, Pietermaritzburg, South Africa).

**Table 1:** Chemical composition of feeds.

Feeds	DM (g/kg)	Ash (g/kg)	EE (g/kg)	ADF (g/kg)	NDF (g/kg)	CP (g/kg)	ADIN (g/kg)	CT (g/kg)
Lucerne hay	967.3	108.0	28.7	304.2	406.4	119.6	9.3	‐
AMF	879.7	44.7	31.8	658.3	753.3	157.0	16.5	31
Maize silage	964.6	56.5	25.8	339.8	607.4	81.0	6.6	‐
*Themeda triandra* hay	940.4	63.3	14.8	522.2	844.1	69.1	5.4	‐
Perrenial ryegrass + white clover	964.5	121.8	18.5	293.1	536.5	219.1	12.6	‐
Control compound feed	980.0	88.5	22.5	110.1	280.7	150	8.8	‐
Pooled experimental compound feed	916.3	64.0	26.5	48.9	163.8	145.3	2.5	‐

Abbreviations: ADF, acid detergent fibre; ADIN, acid detergent insoluble nitrogen; AMF, *Acacia mearnsii* forage; CP, crude protein; CT, condensed tannins; DM, dry matter; EE, ether extract; NDF, neutral detergent fibre.

**Table 2:** Ingredients of the experimental compound feed enriched with tannin extract.

	Dietary inclusions of the tannin extract (%)^a^		
Item (kg)	3	1.5	0.75
Crushed maize	498	498	498
Hominy chop	275	275	275
Wheat bran	100	100	100
Molasses liquid	50	50	50
Soya oil cake	30	30	30
Urea	15	15	15
Lime powder	12.5	12.5	12.5
Salt	10	10	10
Monocalcium phosphate 21	5	5	5
Mineral and vitamin premix	5	5	5
Tannins extract	44	22	11

^a^ $\text{Tannin inclusion}\;=\frac{\text{tannin extract}\;\left(\text{kg}\right)\times 68\%\text{tannin extract purity}}{988\;\text{kg of compound feed}}.$

A total of six replicates (*n* = 6) for each of the abovementioned feeds were oven‐dried overnight (60°C), milled (2‐mm sieve) and transferred (2 g) into labelled and weighed nylon bags (a bag size of 18 × 8 cm and a pore size of 40–60 μm). Triplicates of bags containing each abovementioned feeds were incubated for 0, 6, 24, 48, 72, 96 and 120 h in each of two fistulated Jersey cows (primaparous and non‐lactating) that were grazing at Ukulinga research farm (University of KwaZulu‐Natal, Pietermaritzburg, 9°24′E and 30°24′S and an altitude of 775 m). Bags containing feeds incubated at 0 h were not incubated in the rumen of fistulated cows and were not presoaked in water. The fistulated cows accessed water ad libitum while their fistulated area was sanitized daily during data collection. Bags containing feeds were grouped per incubation period and sequentially added as one bundle. For rumen disturbance prevention, all bags containing feeds were removed simultaneously from the rumen. Incubated bags containing feeds including the 0 h bags were washed with tap water until the water was clear. An automated washing machine was used to further wash bags for 30 min (six cycles with each cycle lasting for 5 min) using cold water. Washed bags were oven‐dried (60°C for 48 h).

### 2.2 Chemical analyses

Feed samples were oven‐dried (60°C for 72 h) and ground (2 mm sieve). Using labelled zip lock bags, feed samples of maize silage, *T. triandra* hay, AMF, lucerne, perennial ryegrass+white clover mixture pasture, commercial compound feed and pooled experimental compound feed were sent for chemical composition analysis to the Feed Analysis Centre of Cedara Agricultural College (Pietermaritzburg, South Africa). Feed samples were analysed for nitrogen content (ID 968.06) using the Leco Truspec nitrogen analyser and nitrogen content was multiplied by 6.25 factor to estimate CP (Leco FP200, LECO) as well as for DM (ID 934.01) and ash (ID 942.05) contents using the AOAC method (AOAC, 2000). The ANKOM 220 Fibre analyser was used to determine acid detergent fibre (ADF) and neutral detergent fibre (NDF) using the filter bag technique (ANKOM Technology, 2011). The Soxhlet Buchi 810 Fat analyser (Soxhlet Buchi) was used to estimate the content of ether extract. Acid detergent insoluble nitrogen (ADIN) was determined according to Van Soest et al. (1991). The AMF and tannin‐enriched compound feed were contained in zip‐lock bags and sent to the MIMOSA extract company (NTE House, Redlands Estate, Pietermaritzburg, South Africa) for tannin content analysis using the SLTC method (SLTC, 1965).

### 2.3 Mathematical calculations and statistical analyses

The degradability of DM for each feed sample was measured using this equation:

$$\text{DM degradability}\;\left(\%\right)=\frac{\text{sample weight × DM}-\text{residue weight}}{\text{sample × DM}}\times 100,$$

The degradability of CP was measured using this equation:

$$\begin{array}{l} \mathrm{CP}\;\mathrm{degradability}\;(\%)=\frac{\text{sample weight × DM}\;\times \;\text{CP}-\text{residue weight}\times \text{CP}}{\text{sample DM}\times \text{CP}}\times 100. \end{array}$$

The DM and CP degradability values were fitted in the following equation to measure the degradation parameters of individual feeds and experimental and control compound feeds: $P=a+b\left(1-e^{-\text{ct}}\right)$ (Ørskov & McDonald, 1979), where *P* is the degradability, *a* is the rapidly degradable fraction, *b* is the insoluble but slowly degradable fraction; *c* is the degradation rate of *b* and *t* is time. The effective degradability (ED) of DM or CP was calculated using the following formula: $\text{ED}(\%)=a+\frac{\left(b\times c\right)}{\left(c+k\right)}$ and *k* is the rate of passage assumed to be 0.03 h^−1^. The effective rumen degradable protein (ERDP) and digestible undegraded protein (DUP) were calculated using the AFRC manual (Alderman & Cottrill, 1996).

All statistical analyses were conducted using SAS software. The rumen DM and CP degradation parameters for inclusions of the tannin extract‐enriched compound feed and the control compound feed; or maize silage, lucerne hay, *T. triandra* hay and perennial ryegrass+white clover mixture pasture were determined using the NLIN procedure. Then the general linear model procedure was used to compare the differences between the rumen degradation parameters of the abovementioned feeds using the following model: $Y_{i}=µ+\alpha_{i}+\varepsilon_{i}$, where $Y_{i}$ is the dependent variable, µ is the overall mean, $\alpha_{i}$ is the treatment effect and $\varepsilon_{i}$ is the experimental error. The results were reported as least square means and pooled standard error(s) of means. Tukey test was used to test the differences between treatment least square means. The contrast statement was used to determine the linear and quadratic effects. For only the experimental compound feeds and the control feed, to measure the relationship between tannin extract inclusion in compound feeds and the DM or CP degradation parameters, orthogonal contrasts were used to measure the linear and quadratic effect. Statistical significance was declared at *p* < 0.05.

## 3 RESULTS

The effect of tannin extract in compound feed on the in sacco degradability kinetics of DM are demonstrated in Table 3. Tannins extract inclusions in compound feed only affected the (*a*) fraction, degradation rate and potential degradability. In response to tannin extract inclusion in compound feed, the (*a*) fraction and degradation rate changed linearly (*p* < 0.05), while the potential degradability and (*a*) fraction changed quadratically (*p* < 0.05).

**Table 3:** Effect of tannin extract in compound feed on the in sacco degradability kinetics of dry matter.

	Tannin extract (%)					Significance		
Item	0	0.75	1.5	3	SEM	GLM	Linear	Quadratic
*a*, %	38.2^b^	44.1^a^	47.2^a^	44.1^a^	1.3	0.0008	0.0034	0.00013
*b*, %	52.7	52.5	48.4	54.6	3.6	0.76732	0.91	0.438
*c*, h^−1^	0.1^a^	0.1^ab^	0.1^ab^	0.1^b^	0.0	0.0395	0.0146	0.62
PD, %	90.9^b^	95.7^ab^	95.7^a^	91.9^ab^	1.4	0.0217	0.659	0.0036
ED, %	78.0	78.2	80.7	75.5	1.6	0.21746	0.549	0.1020

*Note*: Different superscripts per row define the significant difference.

Abbreviations: *a*, rapidly degradable fraction; *b*, undegradable but potentially degradable fraction; *c*, degradation rate of *b*; ED, the effective degradability, assuming *k* of 0.03 h^−1^; GLM, general linear model; PD, potential degradability which is *a* + *b*; SEM, standard error of means.

Significant difference, *p* < 0.05.

The effect of varying tannin extract inclusions in compound feed on the in sacco CP degradation kinetics is demonstrated in Table 4. Tannin inclusions in compound feed only affected the (*a*) and (*b*) fractions and the potential degradability (*p* < 0.05). The (*a*) and (*b*) fractions, as well as the potential degradability changed linearly (*p* < 0.05) to tannin extract inclusions, while the (*a*) fraction, potential degradability and ED changed quadratically (*p* < 0.05) to tannin extract inclusions.

**Table 4:** The effect of varying tannin extract inclusions in compound feed on the in sacco degradation kinetics of crude protein.

	Tannin extract (%)					Significance		
Item	0	0.75	1.5	3	SEM	GLM	Linear	Quadratic
*a*, %	39.4^b^	49.6^a^	51.7^a^	49.9^a^	1.1	<0.0001	<0.0001	<0.0001
*b*, %	51.6^a^	48.1^ab^	44.4^b^	47.2^ab^	1.67	0.05	0.03	0.07
*c*, h^−1^	0.1	0.1	0.1	0.1	0.01	0.13	0.12	0.67
PD, %	91.1^b^	96.0^a^	96.1^a^	95.1^a^	1.0	0.01	0.01	0.03
ED, %	78.5	79.9	83.5	78.7	1.4	0.08	0.53	0.04
ERDP, %	10.6	10.2	10.6	9.1	0.2	0.11	0.17	0.61
DUP, %	1.50	1.2	1.3	1.3	0.1	0.17	0.22	0.08

*Note*: Different superscripts per row define the significant difference.

Abbreviations: *a*, rapidly degradable fraction; *b*, undegradable but potentially degradable fraction; *c*, degradation rate of *b*; DUP, rumen digestible undegradable protein; ED, the effective degradability, assuming *k* of 0.03 h^−1^; ERDP, effective rumen degradable protein; GLM, general linear model; PD, potential degradability which is *a*+*b*; SEM, standard error of means.

Significant difference, *p* < 0.05.

The in sacco DM degradation kinetics of AMF compared to conventional forages are demonstrated in Table 5. Feed type affected (*p* < 0.05) the (*a*) and (*b*) fractions, potential degradability and effective degradability (ED). *T. triandra* hay and AMF had the least (*a*) fraction while lucerne, maize silage and perennial ryegrass+white clover mixture pasture had similar and highest (*a*) fractions. The AMF had the least (*b*) fraction while perennial ryegrass+white clover mixture pasture had the highest (b) fraction. The AMF had the least potential degradability and ED while the perennial ryegrass+white clover mixture pasture had the highest potential degradability and ED. The feed type did not affect (*p* > 0.05) the degradation rate.

**Table 5:** In sacco dry matter degradation kinetics of *Acacia mearnsii* forage (AMF) compared to dairy roughages.

							Significance
Items	Lucerne	AMF	Maize silage	Perrenial ryegrass + white clover	*Themeda triandra* hay	SEM	Feed type
*a*, %	41.5^a^	8.1^b^	39.7^a^	42.8^a^	11.3^b^	1.7	<0.0001
*b*, %	34.7^b^	14.6^c^	41.1^ab^	49.9^a^	42.2^ab^	2.4	<0.0001
*c*, h^−1^	0.1	0.2	0.1	0.1	0.03	0.5	0.29
PD, %	76.2^b^	23.6^d^	80.8^b^	92.7^a^	53.4^c^	2.3	<0.0001
ED, %	69.1^b^	18.1^d^	71.2^b^	81.4^a^	32.8^c^	1.7	<0.0001

*Note*: Different superscripts per row define the significant difference.

Abbreviation: *a*, rapidly degradable fraction; *b*, undegradable but potentially degradable fraction; *c*, degradation rate of *b*; ED, the effective degradability, assuming *k* of 0.03 h^−1^; PD, potential degradability which is *a* + *b*; SEM, standard error of means.

Significant difference, *p* < 0.05.

The in sacco CP degradation kinetics of AMF compared to conventional roughages are demonstrated in Table 6. The feed type affected (*p* < 0.05) the (*a*) and (*b*) fractions, potential degradability, ED and DUP. *T. triandra* hay and AMF had similar, but the least (*a*) fraction, while lucerne, maize silage and perennial ryegrass+white clover mixture pasture had similarly the highest (*a*) fraction. The AMF had the least (*b*) fraction, while lucerne, maize silage, perennial ryegrass+white clover mixture pasture and *T. triandra* hay had the highest (*b*) fraction. The AMF had the least potential degradability and ED, while lucerne, maize silage, perennial ryegrass+white clover mixture pasture had the highest potential degradability and ED. The DUP was the highest in AMF and similarly the least in conventional dairy feeds. The degradation rate was not affected by feed type (*p* > 0.05).

**Table 6:** In sacco crude protein degradation kinetics of *Acacia mearnsii* forage (AMF) compared to dairy roughages.

							Significance
Items	Lucerne	AMF	Maize silage	Perrenial ryegrass + white clover	*Themeda triandra* hay	SEM	Feed type
*a*, %	44.6^a^	19.9^b^	41.8^a^	41.6^a^	16.5^b^	1.8	<0.0001
*b*, %	31.1^a^	12.8^b^	39.7^a^	44.6^a^	39.7^a^	4.1	0.0001
*c*, h^−1^	0.1	0.2	0.1	0.01	0.03	0.1	0.22
PD, %	75.8^a^	32.8^c^	81.5^a^	86.2^a^	56.2^b^	3.5	<0.0001
ED, %	70.1^a^	28.7^b^	72.2^a^	74.7^a^	36.8^b^	2.5	<0.0001
DUP, %	1.7^b^	9.6^a^	1.4^b^	3.0^b^	2.9^b^	6.1	<0.0001

*Note*: Different superscripts per row define significant difference.

Abbreviations: *a*, rapidly degradable fraction; *b*, undegradable but potentially degradable fraction; *c*, degradation rate of *b*; DUP, digestible undegraded protein; ED, the effective degradability, assuming *k* of 0.03 h^−1^; PD, potential degradability which is *a* + *b*; SEM; standard error of means.

Significant difference, *p* < 0.05.

## 4 DISCUSSION

The present study showed lower (*a*) fraction of DM for the control compared to tannin‐enriched compound feeds. This observation must have been due to the tannin inclusion effect on fibrolytic bacteria species. Fibrolytic bacterial species increment have been noted in tannin‐enriched concentrate (Vasta et al., 2010). Additionally, increments of *Ruminococcus albus*, *Ruminococcus flavefaciens* and *Fibrobacter succinogenes* have been observed from tanniferous feed inclusions, *Leucaena leucocephala* and *Morchella esculenta* (Harun et al., 2017). The current study's similar observation of the tannin inclusion effect on the (*a*) fraction and potential degradability in DM and CP ascribes to the proportional relationship that exists between the degradation kinetics of DM and CP (Aufrere et al., 2014).

The present study demonstrated no effect on the (*b*) fraction, but reduced degradation rate by tannin extract inclusions in compound feed. Various rumen bacterial species aid in ruminal fibre digestion, which may aid in maintaining ruminal fibrolysis when tannin‐sensitive fibrolytic microbial species decrease (Saminathan et al., 2015). Therefore, tannins may have no impact on the fibre digestion but may affect fibre digestion rate by reducing microbial diversity in fibrolytic bacteria. Dietary tannin addition has been linked to reduced cellulose and hemicellulose content during fermentation as well as microbial species diversity (Díaz Carrasco et al., 2017).

As a result, DM potential degradability (*a* + *b*) might have increased due to increased (*a*) fraction, as the (*b*) fraction remained unchanged in the present study. Increased proteolytic activity over fibrolytic activity has been seen in rumen microbes (Saminathan et al., 2016). The notion that tannin inclusion increased proteolytic bacteria in the present study is supported by increased (*a*) fraction and potential degradability without changing degradation rate of the (*b*) fraction. Since the incidence of the tannin‐protein complex rises with dietary tannin inclusions, the current study's reduced (*b*) fraction of CP might be due to by‐pass protein.

The present study demonstrated that the (*a*) fraction of tannin inclusions in compound feed increased in the treatment group from the control, but this variable was the same among treatment groups. Concentrate digesting microbes increase when mainly concentrate diets are consumed due to protein substrate presence in the rumen. Tannin supplementation of soyabean has already been shown to improve *B. solvens*, methanogens, total bacteria and microbial crude protein (Phesatcha et al., 2022). Tannins also improve protein digestibility by reducing protozoa populations, which have been shown to engulf rumen bacterial populations (Mohammadabadi & Jolazadeh, 2017). Furthermore, tannins boost rumen microbial populations by increasing their growth factor, ammonia‐nitrogen (ammonia‐N) (Barros‐Rodríguez et al., 2015).

The current study found that AMF has poorer rumen DM and CP degradability kinetics compared to the conventional dairy feeds. Tanniferous sources are notorious for low degradation kinetics. For instance, rumen degradation kinetics outperform in lucerne than in sainfoin (Aufrère et al., 2014); in perennial ryegrass than in chicory (Sun et al., 2012) or in maize silage than in *Terminalia chebula* inclusions (Dhakal et al., 2022). The present study's similarity of low DM's (*a*) fraction in AMF and hay might have been due to low protein content in both feed types as forage to concentrate ratio has an inverse relationship with DM degradability (Jadhav et al., 2018). The inferior AMF's (*b*) fraction to dairy feeds in the current study may be due to low fibre digestibility since browse trees contain lignin, which is less digestible (Basha et al., 2015).

The current study's lower (*b*) fraction and ED of DM in AMF compared to dairy feeds could have been due to low activity of fibrolytic bacteria due to the tannin and lignin content. Acacia species such as AMF contain more structural components in the rumen such as acid detergent lignin, ADF, NDF than convectional feeds (Terefe et al., 2018). The NDF, ADF or acid detergent lignin content have a greater negative impact on the (*a*) or (*b*) fraction, degradation rate, PD and ED of DM or CP than tannin content (Basha et al., 2015).

Previous results show that tannin supplements reduce ruminal bacteria (Fitri et al., 2022) whereas laccase enzyme supplementation increases them (Yue et al., 2020). In tannin sources, the association between the degradation rate and tannin content is moderately negative (Basha et al., 2015). This suggests that, to some extent, the change in the degradation rate by AMF was due to the tannin content, but the inferior protein content combined with indigestible fibre in this feed must have also played a role (Abdullah et al., 2018). Additionally, low crude protein degradability in AMF must have restricted proteolytic bacteria activity due to low protein content and high fibre content, which may have reduced ammonia‐N production.

Ammonia‐N is associated with proteolytic bacteria growth, such as *S. bovis* (Santos et al., 2020). However, the existence of structural cell wall in conjunction with tannins in AMF must have hindered proteolytic bacteria growth in the current investigation, as evidenced by low DUP in AMF. High dietary structural cell wall content decreases protein digestibility (Wanapat et al., 2014), while tannins reduce ammonia‐N (Suescun‐Ospina et al., 2022). However, degradability of Acacia species increases in early and late wet season (Basha et al., 2015), as ADF, ADL or NDF decrease in the wet season than in dry season (Melaku et al., 2010). Therefore, opting for employing AMF harvested in the wet season for the present study might have shown enhanced DM and CP degradation.

## 5 CONCLUSIONS

Tannin extract inclusions in compound feed changed the (*a*) fraction, potential degradability, and the degradation rate of DM, as well as the (*a*) fraction, (*b*) fraction, PD of CP degradation. There was a linear change in the (*a*) fraction and degradation rate of DM, and quadratic changes in the (*a*) fraction and PD of CP degradation. In terms of CP degradation, (*a*) and (*b*) fractions, and PD changed linearly, while the (*a*) fraction, PD and ED changed quadratically. Furthermore, as compared to dairy feeds such as maize silage, *T. triandra* hay, perennial ryegrass+white clover mixture pasture and lucerne hay, AMF had poor DM and CP degradation kinetics.

## CONFLICT OF INTEREST STATEMENT

The authors declare no conflict of interest.

## Data Availability

Data are available upon request.
